# Aberrant spontaneous static and dynamic amplitude of low‐frequency fluctuations in cerebral small vessel disease with or without mild cognitive impairment

**DOI:** 10.1002/brb3.3279

**Published:** 2023-10-10

**Authors:** Xulian Zhang, Zhigang Wang, Darui Zheng, Xuan Cao, Wenzhang Qi, Qianqian Yuan, Da Zhang, Xuhong Liang, Yiming Ruan, Shaojun Zhang, Weijie Tang, Qingling Huang, Chen Xue

**Affiliations:** ^1^ Department of Radiology Nantong Haimen District People's Hospital Nantong China; ^2^ Department of Radiology The Affiliated Brain Hospital of Nanjing Medical University Nanjing China; ^3^ Division of Statistics and Data Science, Department of Mathematical Sciences University of Cincinnati Cincinnati Ohio; ^4^ Department of Statistics University of Florida Gainesville Florida; ^5^ Anhui Conch Cement Company Limited Wuhu China

**Keywords:** amplitude of low‐frequency fluctuation, cerebral small vessel disease, dynamic, mild cognitive impairment, static

## Abstract

**Background:**

Cerebral small vessel disease (CSVD) is considered an age‐related degenerative neurological disorder and the most common risk factor for vascular cognitive impairment (VCI). The amplitude of fluctuation of low frequency (ALFF) can detect altered intrinsic brain activity in CSVD. This study explored the static and dynamic ALFFs in the early stage of CSVD with (CSVD‐M) or without (CSVD‐W) mild cognitive impairment (MCI) in these patients and how these changes contribute to cognitive deterioration.

**Methods:**

Thirty consecutive CSVD cases and 18 healthy controls (HC) were included in this study. All the participants underwent a 3D magnetization‐prepared rapid gradient‐echo (MPRAGE) sequence to obtain structural T1‐weighted images. Simultaneous multislice imaging 5(SMS5) was used for resting‐state functional MRI (rs‐fMRI), and Data Processing and Analysis of Brain Imaging software helped determine static ALFF (sALFF). The dynamic ALFF (dALFF) was calculated using the sliding window method of DynamicBC software. Analysis of Covariance (ANCOVA) and two‐sample *t*‐test were used to evaluate the sALFF and temporal variability of dALFF among the three groups. The subjects were rated on a broad standard neuropsychological scale. Partial correlation analysis was used to evaluate the correlation between sALFF and dALFF variability and cognition (Bonferroni correction, statistical threshold set at *p* < .05).

**Results:**

Compared with HCs, the CSVD‐M group indicated decreased sALFF values in the bilateral cerebellum posterior lobe (CPL) and the left inferior Parietal Lobule (IPL), with increased sALFF values in the right SFG. For dALFF analysis, the CSVD‐W group had significant dALFF variability in the right fusiform gyrus compared with HC. Moreover, the postcentral gyrus (PoCG) was significantly high in the CSVD‐W group. While in the CSVD‐M group, the bilateral paracentral lobules (PL) revealed significantly elevated dALFF variability and low dALFF variability in the left CPL and right IPL compared with HCs. The CSVD‐M group had high dALFF variability in the bilateral PL but low dALFF variability in the left middle temporal gyrus (MTG) and right PoCG compared with the CSVD‐W group. The partial correlation analysis indicated that dALFF variability in the left MTG was positively associated with EM (*r* = 0.713, *p* = .002) in CSVD‐W and CSVD‐M groups. In the groups with CSVD‐M and HC, altered dALFF variability in the bilateral PL was negatively correlated with EM (*r* = −0.560, *p* = .002).

**Conclusion:**

There were significant changes in sALFF and dALFF variability in CSVD patients. Abnormal spontaneous static and dynamic ALFFs may provide new insights into cognitive dysfunction in CSVD with MCI and may be valuable biomarkers for early diagnosis.

## 1 INTRODUCTION

Cerebral small vessel disease (CSVD) is an age‐related degenerative neurological disorder with symptoms like stroke, cognitive impairment, dementia, depression, and gait disturbances (Shi & Wardlaw, 2016). CSVD is considered the most common risk factor for vascular cognitive impairment (VCI) (Cannistraro et al., 2019). VCI indicates cognitive deficits of varying degrees due to blood vessels and their related factors, ranging from subjective cognitive decline and mild cognitive impairment (MCI) to dementia (van der Flier et al., 2018). The clinical diagnosis primarily relies on neuroimaging features because of the insipid onset and slow development of CSVD. CSVD has typical neuroimaging features, including recent small subcortical infarcts, white matter hyperintensity, lacunes, perivascular space, cerebral microhemorrhage, and cerebral atrophy (Wardlaw et al., 2013; Zanon Zotin et al., 2021). The VCI induced by CSVD primarily involves executive function, episodic memory, information processing speed, and attention (Liu et al., 2019). Early identification and timely intervention of VCI due to CSVD can delay disease progression and prevent dementia occurrence (Sanford, 2017). Therefore, finding more sensitive biomarkers for early diagnosis of CSVD with cognitive impairment is essential.

Previous studies have revealed that the amplitude of fluctuation of low frequency (ALFF) is an effective resting‐state functional magnetic resonance imaging (rs‐fMRI) method to assess spontaneous brain activity (Li et al., 2021; Zang et al., 2007). ALFF values have been positively correlated with changes in spontaneous neural activity, widely used to demonstrate spontaneous brain activity within many neurological diseases (Cui et al., 2020; Feng et al., 2021; Hou et al., 2014; Liu et al., 2021). Previous studies on the static ALFF (sALFF) method showed that spontaneous changes in brain activity in CSVD patients are associated with brain dysfunction. Moreover, ALFF abnormalities in specific brain regions are significantly related to cognitive scores in CSVD patients (Feng et al., 2021; Hu et al., 2022; Li et al., 2021; Zhou et al., 2020), potentially serving as sensitive biomarkers depicting the neurological disease pathogenesis (Liang et al., 2014; Ren et al., 2016). A study using ALFF, fractional ALFF, and regional homogeneity methods depicted that altered spontaneous brain activity was related to neurologic dysfunction in CSVD. Additionally, increased sALFF values in the putamen, right insula, and left precuneus decreased sALFF in postcentral and right precentral gyrus in CSVD patients were also observed. The abnormal sALFF was correlated with the cognitive scores, indicating that sALFF change could provide a useful diagnostic index for early CSVD (Feng et al., 2021). Another study on vascular MCI (vascular MCI, vMCI) identified that the vMCI group patients had reduced sALFF values in the bilateral precuneus, angular gyrus, and medial frontal gyrus. Thus, sALFF abnormalities in these brain regions could reflect the underlying mechanism of CSVD cognitive decline (Li et al., 2021). Therefore, measuring sALFF can reveal changes in brain activity in the early disease stages.

Interestingly, previous studies mainly focused on the static and dynamic characteristics of the spontaneous brain and neural activities over time that was ignored by the sALFF analysis method (Li et al., 2021). Recently, a dynamic ALFF (dALFF) method combining ALFF with the sliding window method has been proposed to describe the ALFF change over time. Compared with sALFF, the dALFF method can more effectively capture the abnormal spontaneous neural activity in the brain (Cui et al., 2020; Kim et al., 2017), widely used in many neuropsychiatric diseases (Cui et al., 2020; Kim et al., 2017; Li et al., 2019; Li et al., 2021). The dALFF analysis reflects the dynamic characteristics of spontaneous brain activity, such as excessive variability (increased dALFF variability) and excessive stability (decreased dALFF variability), indicating a certain extent of brain function impairment (Cui et al., 2020; Kim et al., 2017; Li et al., 2019; Li et al., 2021). However, whether CSVD with or without cognitive decline demonstrates abnormal temporal variability in spontaneous brain activity remains unknown. Identifying these abnormal dynamic features will enhance understanding of the neuropathological mechanisms in CSVD. Previous studies with the sALFF and dALFF methods have indicated their consistency in recording brain activity. The combined approach helps clarify the underlying mechanisms of CSVD by analyzing the correlation between sALFF and dALFF variability and cognitive function (Li et al., 2021; Yang et al., 2020).

This study used sALFF and dALFF methods to explore the spontaneous brain activity changes in CSVD and their relationship with cognitive function. We hypothesized that (1) sALFF and dALFF variability varied in the CSVD‐W and CSVD‐M patients; (2) changes in the sALFF and dALFF variability could be associated with neurocognitive scores; (3) the abnormal sALFF and dALFF variability in some brain regions could provide novel potential biomarkers to distinguish CSVD‐M from CSVD‐W.

## 2 MATERIALS AND METHODS

### 2.1 Subjects

Forty‐one CSVD nondementia subjects were recruited from the outpatient and inpatient departments of the Affiliated Brain Hospital of Nanjing Medical University, and 20 healthy controls (HCs) were recruited from the community population between November 2020 and March 2022. All subjects underwent MRI examinations, including T1‐weighted imaging, T2‐weighted imaging, diffusion‐weighted imaging (DWI), a reduced apparent diffusion coefficient (ADC), fluid‐attenuated inversion recovery (FLAIR) images, and three‐dimensional time‐of‐flight magnetic resonance angiography (MRA). However, 13 participants were excluded due to intracerebral lesions (4 CSVD‐M subjects and 1 CSVD‐W subject with meningioma or history of traumatic brain injury) and excessive head movement (4 CSVD‐M subjects, 3 CSVD‐W subject, and 1 healthy control subject cumulative translation or rotation > 2.5 mm or 2.5°) not satisfying the inclusion criteria. Therefore, 48 subjects (18 in the HCs and 30 in the CSVD group) were included in this study. Based on the assessment of the cognitive level by the neuropsychological scale, the CSVD group subjects were further divided into the MCI (*n* = 19) and the nonMCI (*n* = 11) groups.

The inclusion criteria for all the patients were as follows: (1) age: 50–80 years old; (2) native Chinese speaker, right‐handed; (3) years of education ≥6 years; (4) no history of consuming psychotropic drugs; and (5) voluntary participation and signed informed consent.

The inclusion criteria of HC were as follows: (1) no memory disease; (2) CSVD exclusion through imaging examination; (3) normal cognitive performance corresponding to age and education; and (4) clinical dementia score (CDR) = 0.

The inclusion criteria of the CSVD‐W group were as follows: (1) no memory disorders; (2) imaging examinations were consistent with the CSVD diagnostic criteria (specific diagnostic criteria for CSVD are provided in the **Supplementary Materials Methods**); (3) normal cognitive performance corresponding to age and education; and (4) CDR = 0.

The inclusion criteria for CSVD‐M were as follows: (1) patients complained or relatives confirmed memory disorders for at least 3 months; (2) imaging examination was consistent with the CSVD diagnostic criteria; (3) impaired objective memory performance (**Supplementary Materials Methods** for the assessment criteria); (4) overall normal cognitive function, but CDR = 0.5; and (5) no dementia.

The exclusion criteria for all the participants were as follows: (1) history of severe brain diseases, including craniocerebral tumors, macrovascular diseases, massive cerebral infarction, cerebral hemorrhage, traumatic brain injury, etc.; (2) history of brain dysfunction, including psychosis, epilepsy, thyroid insufficiency, etc.; (3) suffering other neurological diseases, including Alzheimer's disease, Parkinson's disease, multiple sclerosis, etc.; (4) major systemic diseases like cancer, severe anemia, syphilis, AIDS, etc.; and (5) patients having contraindicatory MRI examinations or could not finish the neuropsychological scale test.

### 2.2 Neurocognitive assessments

All the participants underwent a comprehensive and standard neuropsychological scale assessment (Fan et al., 2021; Lei et al., 2022; Li et al., 2021; Teng et al., 2022). The Mini‐Mental State Assessment (MMSE) and Montreal Cognitive Assessment (MoCA) helped assess the global cognitive level. The Trail Making Test B (TMT‐B) and Digit Span Test (such as the DS forward and backward) evaluated the executive and attention functions for characteristic cognitive impairment. The Auditory Verbal Learning Test (AVLT), the Rey‐Osterrich Complex Figure Test‐20 min‐delayed recall (CFT‐20‐min DR), and the Logical Memory Test (LMT) helped assess the episodic memory function. The Rey‐Osterrich Complex Figure Test (CFT) determined visuospatial function. The Verbal Fluency Test (VFT) and the Boston Naming Test (BNT) were used to assess language function. Additionally, the CDR evaluated the presence of dementia, and the Hamilton Depression Rating Scale (HAMD) determined concomitant depressive symptoms. Two senior clinical neuropsychologists evaluated these scales.

### 2.3 Image acquisition

Imaging data were acquired using a 3.0T magnetic resonance scanner (Siemens, PRISMA, Germany) and a 64‐channel head coil from the Department of Radiology, Nanjing Brain Hospital. High‐resolution T1WI images were captured using a 3D magnetization‐prepared rapid gradient‐echo (MPRAGE) sequence. These were the specific parameters: repetition time (TR) = 1900 ms, echo time (TE) = 2.52 ms, inversion time (TI) = 1100 ms, flip angle (FA) = 7°, the field of view (FOV) = 256 mm × 256 mm, slice thickness = 1 mm, voxel size = 1.0 × 1.0 × 1.0 mm^3^, and the number of slices = 176. The simultaneous multislice imaging 5 (SMS5) helped obtain the functional imaging data with the following scan parameters (Barth et al., 2016): TR = 500 ms, TE = 30 ms, slice thickness = 4 mm, number of slices = 36, voxel size = 3.1 × 3.1 × 4.0 mm^3^, FA = 60°, FOV = 220 mm × 220 mm; 960 time points, and about 8 min of scanning duration. The patients also underwent T2 fluid‐attenuated inversion recovery, diffusion‐weighted imaging, and quantitative susceptibility mapping. During data collection, subjects were instructed to stay awake with closed eyes and remain as still as possible. Cotton plugs were used to reduce noise effects, and foam cushion was used for head fixation to decrease motion artifacts.

### 2.4 Image preprocessing

All the fMRI data were preprocessed using MATLAB2014a and Data Processing and Analysis for Brain Imaging (DPABI; http://rfmri.org/dpabi, version 6.1), based on Statistical Parametric Mapping (SPM) (Yan et al., 2016). The detailed steps were (Xue et al., 2021) as follows: (1) removing the first 10 time points to ensure the stability of the MRI signal; (2) correcting time level and head and excluding subjects with excessive head movement (cumulative translation or rotation > 2.5 mm or 2.5°) during scanning; (3) noise covariates were regression processed, and multiple linear regression helped remove noise covariates (such as whole brain signals, cerebrospinal fluid, white matter noise, and 24 head‐movement parameters); (4) the functional images were spatially normalized to the standard space of the Montreal Neurological Institute and resampled using a voxel size of 3 × 3 × 3 mm^3^; (5) scalp removal helped reduce the influence of irrelevant structures on registration; (6) Gaussian kernel having a full width at half‐maximum of 6 mm was used for spatial smoothing; and (7) linear trend removal.

### 2.5 sALFF and dALFF calculation

The static ALFF (sALFF) was computed with the DPABI software. The time series of each voxel was transformed in the frequency domain, and a fast Fourier transform obtained the power spectrum. The square root was measured at each power spectrum frequency, and the average square root, that is, ALFF value, was acquired [0.01–0.08Hz range]. The ALFF map obtained was normalized, and each voxel was divided by the average ALFF value of the whole brain signal to secure the mALFF spatial map.

The DynamicBC toolbox (http://restfmri.net/forum/DynamicBC) was used to calculate dALFF (Cui et al., 2020). Based on previous studies (Liao et al., 2014), the classical sliding time window method was selected, taking 200TRs (100s) as the window length and 2TRs (1s) as the step length for obtaining 376 Windows (Cui et al., 2020; Ma et al., 2020; Zheng et al., 2021). Within each frequency band ([0.01–0.08 Hz]) window(Li et al., 2021) for each subject, the ALFF map calculation was divided by the global mean ALFF value for each window, providing a series of dynamic ALFF maps. Finally, the dALFF map variance over time helped measure the temporal variability of intrinsic brain activity.

### 2.6 Statistical analyses

Statistical analyses were performed using the Statistical Package for the Social Sciences (SPSS) software version 22.0 (IBM, Armonk, New York, NY, USA). The analysis of covariance (ANCOVA) and the chi‐square test (for gender) helped compare the demographic, neurocognitive and gray matter volumes data among the three groups, including the HC, CSVD‐W, and CSVD‐M. Bonferroni correction was used for post hoc comparison by a two‐sample *t*‐test with a *p* < .05 significant level. Two‐sample rank‐sum test was used to compare the total imaging burden and imaging markers between CSVD‐W and CSVD‐M, Mann–Whitney *U* correction, *p* < .05.

We used DPABI software to extract the gray matter (GM) volume of each subject; after controlling for age, sex, education level, and GM volume, ANCOVA compared the differences in dALFF and sALFF among the three groups by selecting a whole brain gray matter mask. The two‐sample *t*‐test method was adopted to conduct post hoc comparisons of the ANCOVA results. Age, gender, education level, and GM volume were covariates. The Gaussian random field (GRF) method was utilized for multiple comparisons (4 mm FWHM Gaussian smoothness, voxel‐*p* < .05, cluster‐*p* < .05).

We used DPABI to extract dALFF variability in brain regions with significant changes in both groups of CSVD patients (including left middle temporal gyrus, right superior temporal gyrus, bilateral paracentral lobule, and the right postcentral gyrus), and then used it for correlation analysis. After controlling for the influence of age, sex, and education level, partial correlation analysis was performed to reveal the relationship between dALFF variability and cognitive domain in CSVD patients (*p* < .05, Bonferroni corrected).

It is worth mentioning that we divided the neurocognitive scale into three cognitive domains commonly involved in CSVD (mainly including executive and attention function, episodic memory, and language function). The performance and attention function data are mainly obtained through DST, MTT‐B, etc. The episodic memory data mainly came from AVLT‐20‐min DR, LMT‐20‐min DR, and CFT‐20‐min DR, while the language function data mainly came from VFT and BNT. The individual raw scores for each neuropsychological test were converted into normalized Z‐scores. Then, the normalized Z‐scores are averaged to calculate the composite Z‐scores for each cognitive domain.

### 2.7 Validation analysis

We performed validation analyses with different window lengths to verify the reliability of the dALFF variability results obtained with a window length of 200TR (100s). A window length of 160TR (80s) was selected to recalculate the main results, as detailed in **Supplementary Material S1**.

## 3 RESULTS

### 3.1 Demographic and neurocognitive characteristics

The demographic information, neurocognitive characteristics, and gray matter volumes of 18 HCs (mean age 61.67 ± 7.62 years), 11 CSVD‐W patients (mean age 66.64 ± 7.06 years), and 19 CSVD‐M patients (mean age 67.89 ± 8.01 years) have been represented in **Table** **1**. The total imaging burden and imaging markers for CSVD‐W and CSVD‐M are shown in **Table** **2**. There were significant differences in age and sex between CSVD and HC groups, but no such difference in the education level and gray matter volumes among the groups. The episodic memory (EM) score of the CSVD‐M group was significantly lower than that of the HC and CSVD‐W groups, and the language function of the CSVD‐M group was significantly lower than that of the CVSD‐W group (Bonferroni's post hoc correction, *p* < .05). There were no significant differences in total imaging burden and imaging markers between CSVD‐W and CSVD‐M groups (Mann–Whitney *U* correction, *p* < .05).

**TABLE 1:** Demographics and clinical measures of three groups, including CSVD‐W, CSVD‐M, and HC.

	HC (18)	CSVD‐W (11)	CSVD‐M (19)	F‐values(χ2)	*p* Values
Age (years)	61.67 ± 7.62	66.64 ± 7.06	67.89 ± 8.01	3.282	.047
Gender (male/female)	6/12	3/8	10/9	2.344	.310
Education level (years)	8.97 ± 2.48	11.32 ± 3.12	8.74 ± 3.12	3.110	.054
MMSE	27.71 ± 1.57	27.91 ± 1.45	26.63 ± 1.61	3.161	.052
MoCA	26.53 ± 0.62	27.18 ± 1.17	23.47 ± 2.01	30.172	<.001^bc^
TMT‐B	195.92 ± 59.65	183.70 ± 39.33	255.82 ± 59.24	5.490	.009^bc^
DST	5.58 ± 0.67	5.75 ± 1.06	4.75 ± 0.86	5.322	.009^bc^
AVLT‐20‐min DR	20.92 ± 1.08	22.30 ± 1.49	17.47 ± 3.23	15.104	<.001^bc^
CFT‐20‐min DR	11.75 ± 4.27	15.90 ± 7.75	6.92 ± 2.81	8.321	.001^c^
LMT	11.69 ± 3.30	13.50 ± 3.13	9.83 ± 4.15	2.881	.071
LMT‐DR	9.77 ± 3.22	11.30 ± 3.50	7.92 ± 3.73	2.613	.089
VFT	15.43 ± 4.03	19.70 ± 4.62	15.20 ± 4.14	4.016	.027^c^
BNT	7.77 ± 0.93	8.20 ± 0.92	6.77 ± 0.93	7.460	.002^bc^
HAMD	2.54 ± 2.40	5.50 ± 4.70	5.00 ± 5.54	1.548	.227
Gray matter volumes	527.02 ± 45.04	496.81 ± 69.47	472.20 ± 94.36	2.575	.087
Composite Z‐scores of each cognitive domain					
Executive & attention function	–0.403 ± 1.208	–0.054 ± 1.316	0.109 ± 1.227	0.533	.592
Episodic memory	0.146 ± 0.450	0.669 ± 0.665	–0.720 ± 0.674	13.902	<.001^bc^
Language function	0.038 ± 0.716	0.669 ± 0.776	–0.491 ± 0.679	7.341	.002^c^

*Note*: Numbers are given as means ± standard deviation, SD unless stated otherwise. Values for age and education derived from ANOVA; gender from chi‐square test; all clinical measures from ANCOVA with age, gender, years of education as covariates.

MMSE, Mini‐Mental State Examination; MoCA, Montreal Cognitive Assessment Test; TMT‐B, Trail Making Test B; DST, Digit Span Test; AVLT‐20‐min DR, Auditory Verbal Learning Test‐20 min‐delayed recall; CFT‐20‐min DR, Rey‐Osterrich Complex Figure Test‐20 min‐delayed recall; LMT, Logical Memory Test; LMT‐DR, Logical Memory Test‐delayed recall; VFT, Verbal Fluency Test; BNT, Boston Naming Test; HAMD, Hamilton Depression Rating Scale; GMV, gray matter volumes.

^a^Post hoc analyses showed a significant group difference between CSVD‐W and HC.

^b^Post hoc analyses showed a significant group difference between CSVD‐M and HC.

^c^Post hoc analyses showed a significant group difference between CSVD‐M and CSVD‐W.

**TABLE 2:** Total imaging burden and imaging markers of CSVD‐W and CSVD‐M.

	Groups	M (P25, P75)	Two‐sample rank‐sum test	
			*Z*	*p*
**Total imaging burden**	CSVD‐W	2.09 (2.00, 2.00)	–0.080	.936
	CSVD‐M	2.11 (2.00, 2.00)		
**Lacunae or new subcortical infarcts**	CSVD‐W	1.91 (0.00, 3.00)	–1.223	.221
	CSVD‐M	1.37 (0.00, 2.00)		
**WMH (Fazekas score)**	CSVD‐W	2.18 (2.00, 2.00)	–0.584	.559
	CSVD‐M	2.11 (2.00, 2.00)		
**CMBs**	CSVD‐W	0.18 (0.00, 0.00)	–1.170	.242
	CSVD‐M	0.84 (0.00, 1.00)		
**Enlarged PVS**	CSVD‐W	6.82 (5.00, 8.00)	–0.238	.812
	CSVD‐M	7.84 (4.00, 13.00)		

*Note*: The above data did not meet the normality test, so the two‐sample rank‐sum test (Mann–Whitney *U* correction) was used and the mean (percentile) was used for description.

WMH, white matter hyperintensity; CMBs, cerebral microbleeds; PVS, perivascular space.

### 3.2 Static and dynamic ALFF changes in CSVD‐M and CSVD‐W groups

**FIGURE 1:**
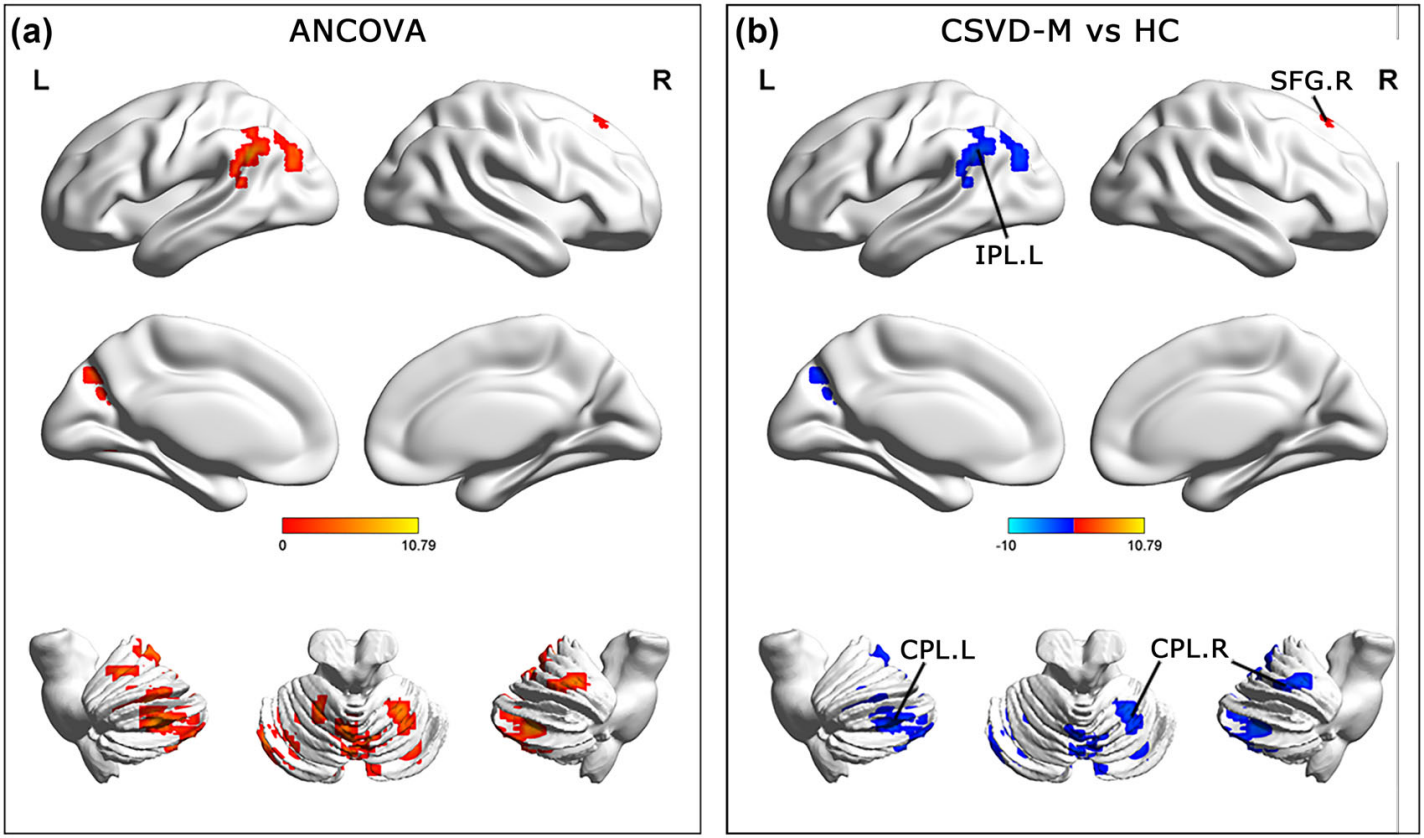
Brain regions exhibiting significant differences in static amplitude of fluctuation of low frequency. **(a)** Brain regions of static amplitude of fluctuation of low frequency showed significant differences among CSVD‐M, CSVD‐W, and HC groups. **(b)** Results of post hoc analyses were performed using two‐sample *t*‐tests. Gaussian random field (GRF) corrected. The voxel level and the clustering level were set to *p* < .05 and *p* < .05, respectively. CSVD‐M, cerebral small vessel disease with mild cognitive impairment; HC, healthy controls; CPL, cerebellum posterior lobe; IPL, inferior parietal lobule; SFG, superior frontal gyrus; R, right; L, left.

ANCOVA results revealed significant differences in brain regions among the three groups for sALFF analysis. These regions were located in the bilateral cerebellum posterior lobe (CPL), left inferior parietal lobule (IPL), and right superior frontal gyrus (SFG). Compared to the HCs, the sALFF value in the right SFG was elevated, and sALFF values in the bilateral CPL and left IPL were reduced in the CSVD‐M group (GRF corrected, 4 mm FWHM Gaussian smoothness, voxel‐*p* < .05, cluster‐*p* < .05) (**Table** **2** and **Figure** **1**).

**FIGURE 2:**
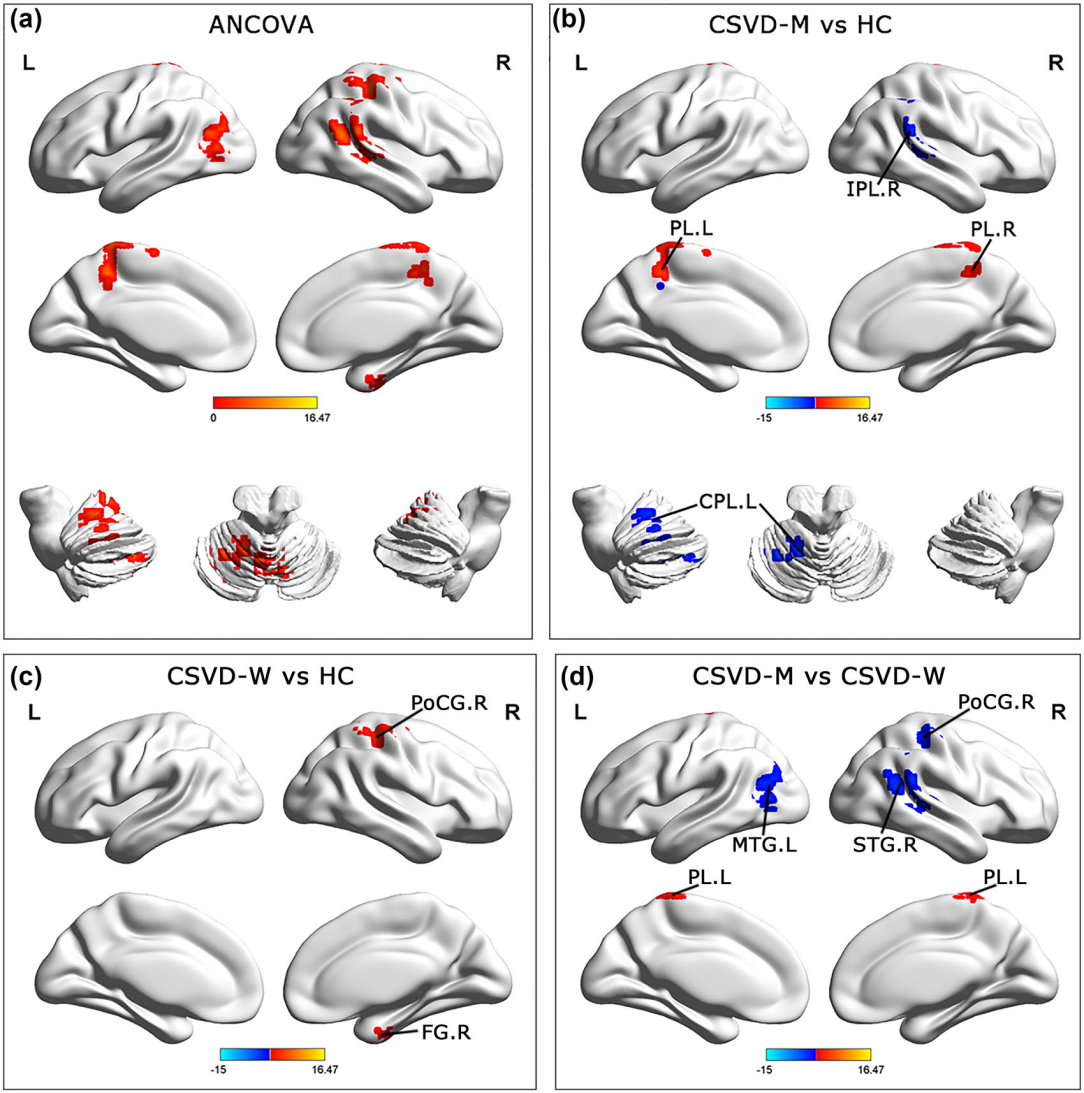
Brain regions exhibiting significant differences in dynamic amplitude of fluctuation of low frequency. **(a)** Brain regions of dynamic amplitude of fluctuation of low frequency showed significant differences among CSVD‐M, CSVD‐W, and HC groups. **(b–d)** Results of post hoc analyses were performed using two‐sample *t*‐tests. Gaussian random field (GRF) corrected. The voxel level and the clustering level were set to *p*< .05 and *p*< .05, respectively. CSVD‐M, cerebral small vessel disease with mild cognitive impairment; CSVD‐W, cerebral small vessel disease without mild cognitive impairment; HC, healthy controls; CPL, cerebellum posterior lobe; IPL, inferior parietal lobule; PL, paracentral lobule; PoCG postcentral gyrus; FG, fusiform gyrus; MTG, middle temporal gyrus; STG, superior temporal gyrus; R, right; L, left.

ANCOVA results also showed significant differences in dALFF variability among the three groups for the dynamic ALFF analysis. These regions were located in the right fusiform gyrus (FG), left CPL, left middle temporal gyrus (MTG), right superior temporal gyrus (STG), bilateral paracentral lobule (PL), and the right postcentral gyrus (PoCG). Compared with HC, the CSVD‐W group revealed increased dALFF values in the right FG and the postcentral gyrus. Moreover, the CSVD‐M group indicated elevated dALFF values in the bilateral PL and decreased dALFF values in the left CPL and right IPL than in HCs. Compared with the CSVD‐W group, the CSVD‐M group had elevated dALFF values in the bilateral PL and decreased dALFF values in the right SFG, left MTG, and right PoCG (GRF corrected, 4 mm FWHM Gaussian smoothness, voxel‐*p* < .05, cluster‐*p* < .05). Age, sex, years of education, and GM volume were covariates for all the results (**Table** **3** and **Figure** **2**).

**TABLE 3:** Differences in static ALFF and dynamic ALFF variability among the three groups.

Region (aal)	Peak MNI coordinate			*F*/*T* values	Cluster number
	*x*	*y*	*z*		
**Static ALFF**					
**ANCOVA**					
B cerebellum posterior lobe	0	–72	–12	10.3752	648
L inferior parietal lobule	–48	–48	30	10.7941	175
R superior frontal gyrus	36	33	48	8.5731	174
**CSVD‐M vs. HC**					
B cerebellum posterior lobe	0	–72	–12	–4.3765	539
R superior frontal gyrus	15	36	54	4.0869	168
L inferior parietal lobule	–18	–66	30	–4.3575	172
**Dynamic ALFF variability**					
**ANCOVA**					
R fusiform gyrus	21	–3	–45	11.8317	116
L cerebellum posterior lobe	–30	–60	–30	16.4656	243
L middle temporal gyrus	–51	–72	12	10.4548	99
R superior temporal gyrus	51	–51	18	8.6249	201
B paracentral lobule	6	–39	51	10.2973	268
R postcentral gyrus	33	–27	48	7.2104	187
**CSVD‐W vs. HC**					
R fusiform gyrus	39	–6	–48	5.6677	102
R postcentral gyrus	39	–30	54	4.7671	183
**CSVD‐M vs. HC**					
L cerebellum posterior lobe	–27	–57	–27	–5.4179	156
R inferior parietal lobule	42	–42	33	–4.1368	130
B paracentral lobule	6	–39	51	4.3856	245
**CSVD‐M vs. CSVD‐W**					
R superior temporal gyrus	51	–45	3	–5.2997	159
L middle temporal gyrus	–51	–72	12	–3.9943	95
R postcentral gyrus	36	–33	66	–3.3559	88
B paracentral lobule	–6	–36	72	4.3963	113

*Note*: The *x*, *y*, *z* coordinates are the primary peak locations in the MNI space. All results are displayed after adjusting for age, sex, and education at a threshold of *p* < .05, GRF corrected.

ALFF, amplitude of low‐frequency fluctuations; HC, healthy controls; CSVD, cerebral small vessel disease; CSVD‐W, CVSD without mild cognitive impairment; CSVD‐M, CVSD with mild cognitive impairment; L, left; R, right; B, bilateral; MNI, Montreal Neurological Institute.

### 3.3 Correlation analysis results in the three groups

**FIGURE 3:**
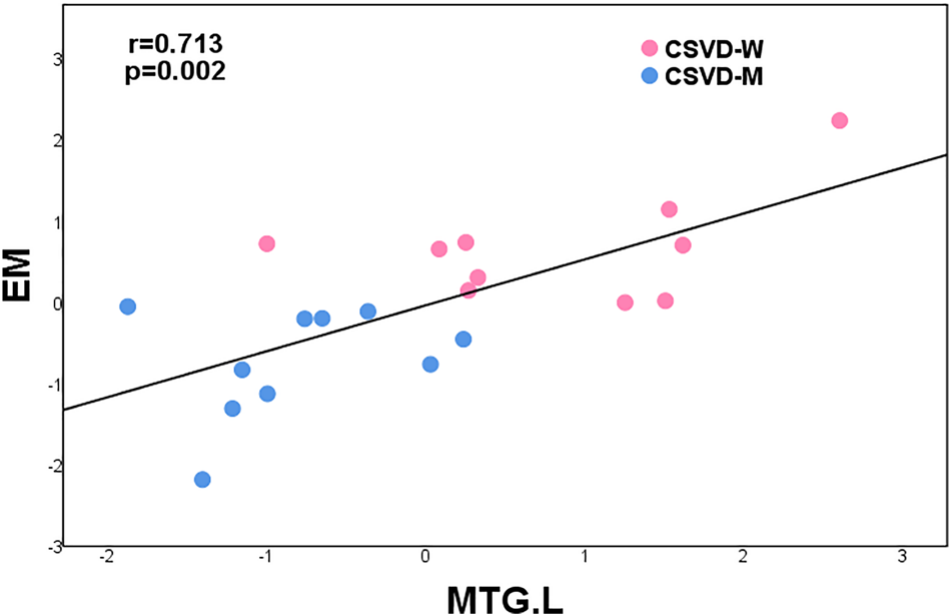
Results of the relationship between dynamic amplitude of fluctuation of low‐frequency variability and cognitive function. There was a significant correlation between dALFF variability and EM in the left MTG in the CSVD‐W and CSVD‐M groups. Age, gender, and years of education were used as covariates of results (Bonferroni corrected, *p* < .05). CSVD‐W, cerebral small vessel disease without mild cognitive impairment; CSVD‐M, cerebral small vessel disease with mild cognitive impairment; EM, episodic memory; MTG, middle temporal gyrus; L, left.

**FIGURE 4:**
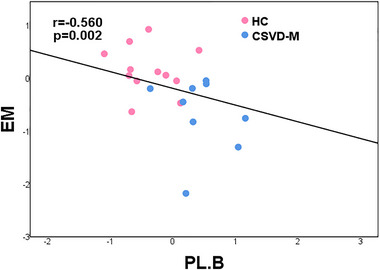
Results of the relationship between dynamic amplitude of fluctuation of low‐frequency variability and cognitive function. There was a significant correlation between dALFF variability and EM in the bilateral PL in the HC and CSVD‐M groups. Age, gender, and years of education were used as covariates of results (Bonferroni corrected, *p* < .05). HC, healthy controls; CSVD‐M, cerebral small vessel disease with mild cognitive impairment; EM, episodic memory; PL, paracentral lobule; B, bilateral.

Partial correlation analysis indicated that dALFF variability in the left MTG was positively associated with EM (*r* = 0.713, *p* = .002) in CSVD‐W and CSVD‐M groups (**Figure** **3**). In the groups with CSVD‐M and HC, altered dALFF variability in the bilateral PL was negatively correlated with EM (*r* = −0.560, *p* = .002) (**Figure** **4**). Age, sex, and years of education were covariates for the results, Bonferroni corrected, *p* < .05.

## 4 DISCUSSION

This study attempted to combine sALFF and dALFF while exploring the strength and stability of spontaneous brain activity in CSVD patients with or without MCI. Significant changes were observed in both sALFF and dALFF in all CSVD patients, corresponding to specific characteristics of spontaneous brain activity. We observed that dALFF variability in left MTG abnormalities was significantly correlated with cognitive performance in the two CSVD groups. These findings highlight the importance of the dynamic characteristics of spontaneous brain activity in CSVD. Moreover, our findings may indicate that abnormal activity within specific brain regions could lead to cognitive impairment.

SALFF value indicates the stability of brain spontaneous activity intensity, and the variability of dALFF reflects the plasticity and flexibility of spontaneous brain activity. The two are unique and complementary, enhancing the understanding of the underlying neuropathological disease mechanisms (Ge et al., 2022). Previous studies have identified that the brain regions with significant changes in ALFF in CSVD patients are mainly the frontal, parietal, temporal, and other regions. Two studies used the ALFF index to observe spontaneous brain activity and observed the elevated ALFF value of the right SFG in CSVD patients (Lei et al., 2014; Zhou et al., 2020). A study of CSVD patients showed a decrease in ALFF in the left superior parietal gyrus (Feng et al., 2021), but we found a decline in ALFF in the inferior parietal lobule. Additionally, previous studies on CSVD rarely reported ALFF changes in the cerebellar gray matter region. However, our study found that both sALFF and dALFF variability in CPL were decreased in CSVD‐M patients.

Our study shows, to a certain extent, that from the rs‐fMRI data from SMS 5 sequence processed by the DPABI software, we can gain insight into the intrinsic brain activity of CSVD with or without MCI. We first conducted sALFF analysis to compare the CSVD‐M group with HCs and identified that sALFF in the CSVD‐M group increased in the right SFG but decreased in the bilateral CPL and left IPL regions. These brain regions with altered activity intensity were primarily associated with the executive (SFG&CPL), language (IPL&CPL), and motor (SFG&IPL) functions. Previous studies have indicated that sALFF increased in the SFG region for CSVD patients (Dong et al., 2021; Ni et al., 2021; Pelkmans et al., 2019; Pena et al., 2019; Ren et al., 2023; Su, Wang et al., 2019; Wang et al., 2015), consistent with our findings. SFG is a core brain region for executing many cognitive tasks and motor control (Li et al., 2013; Wang et al., 2019). The increased spontaneous brain activity in SFG may suggest that compensatory mechanisms in the brain of CSVD‐M patients are compensating for the clinical cognitive deterioration (Ter Telgte et al., 2018).

In contrast, CSVD‐M patients had reduced brain activity in bilateral CPL and left IPL than in HCs. IPL brain regions mainly include the superior marginal and angular gyrus, which primarily correlated with language processing and fine motor control (Merchant et al., 2020; Montefinese et al., 2021). Many studies have reported that the cerebellum is important in motor and coordination control and cognitive and emotional regulation (Li et al., 2021; Stoodley & Schmahmann, 2010; Su et al., 2019). When CPL is affected, it causes cerebellar cognitive and affective syndromes, such as executive and language dysfunction (Hoche et al., 2018; Schmahmann, 2019). Decreased executive function is one of the typical clinical features of CSVD (Ter Telgte et al., 2018), we hypothesize that the reduced brain activity in bilateral CPL and left IPL in CSVD‐M patients may contribute to the impairment of executive and language function in CSVD patients.

Subsequently, dALFF variability was evaluated to describe the spontaneous brain activity over time in CSVD patients and investigate possible ALFF disturbances. Moreover, significant changes in dALFF variability were observed in both groups of CSVD patients. Specifically, CSVD‐W patients had increased dALFF variability in the right FG and PoCG than in HCs. The FG is integral to semantic memory and recognition‐related pathways (Chen et al., 2022; Forseth et al., 2018). Jung et al. (2021) have also observed that gray matter volume in the FG is associated with emotion recognition ability. PoCG is a core brain region of the somatosensory network with a critical role in processing sensory information (Kropf et al., 2019). A preliminary study revealed that PoCG was one of the abnormal cortical regions in the whole brain functional connectivity of MCI and AD (Chen, 2021). Another cortical morphological study in CSVD with subcortical ischemic depression identified a significant elevation of PoCG cortical thickness and complexity, demonstrating complex brain activity (Zhou et al., 2021). Therefore, the dALFF variability in the right FG and PoCG depicts abnormal temporal fluctuations in spontaneous activity within these regions and, therefore, the increased dALFF variability of right FG and PoCG, which may indicate abnormal temporal fluctuations in brain activity in these brain areas. We speculate that frequent changes in brain activity may counter the deterioration of cognitive function and clinical symptoms.

Besides, the CSVD‐M group had increased dALFF variability in bilateral PL and decreased dALFF variability in left CPL and right IPL than in HCs. PL is an extension of the precentral and postcentral gyrus associated with the sensory and motor functions of the lower limbs (Cho et al., 2022). Mascalchi et al. (2014) observed that the increased variability of spontaneous brain activity in PL could be due to the microstructure damage of the corpus callosum in CSVD patients with MCI. The dALFF variability reflects dynamic characteristics of spontaneous brain activity over time, unlike sALFF, which only reflects static changes (Liu et al., 2021). The overall amplitude (sALFF) and variability (dALFF) of CPL and IPL were abnormally reduced. Combined with the results of sALFF analysis, we speculate that the intensity of spontaneous brain activity in these two brain regions is reduced, has little fluctuation over time, and may play an important role in the decline of cognitive function in CSVD patients.

Compared to CSVD‐W patients, dALFF variability in bilateral PL significantly increased, indicating the instability of spontaneous brain activity in CSVD‐M patients in bilateral PL. Meanwhile, CSVD‐M patients had decreased dALFF variability in the right STG and left MTG, and both regions were primarily involved in speech recognition, semantic memory, and visual information processing (Bhaya‐Grossman & Chang, 2022; Xue et al., 2019). Language and memory disorders are essential manifestations of CSVD (Ter Telgte et al., 2018). Studies have revealed that CSVD patients have reduced spontaneous brain activity at MTG (Yang et al., 2020). Furthermore, temporal changes in spontaneous brain activity in these brain regions may partly explain the clinical symptoms of CSVD patients.

Additionally, the dALFF value of left MTG was positively associated with EM in two CSVD groups, consistent with previous studies (Ni et al., 2016; Wang et al., 2019), in the CSVD‐M and HC groups, dALFF values of bilateral paracentral lobule were negatively correlated with EM. This may imply that reduced dALFF variability in the left medial temporal gyrus plays an important role in the episodic memory of patients with CSVD, and the impaired function of bilateral paracentral lobule leads to the impaired episodic memory, but the validity of this conclusion needs to be verified by more studies.

Notably, the CSVD‐W group performed best on several tests, outperforming the HC group. Ter Telgte et al. identified that CSVD patients with similar MRI loads had huge variability in clinical symptoms. This could be due to the brain reserve existence (including structural or functional indicators, such as brain volume), cognitive reserve (referring to lifelong experiences like education), and compensation mechanism. Thus, some CSVD patients can maintain their cognitive function at predisease levels. The CSVD‐W patients should especially belong to the large cognitive reserve that supports the cognitive function of this group at a high level.

MRI‐based CSVD burden assessment is a significant predictor for cognitive impairment. We explored whether sALFF/dALFF, other than conventional imaging markers, could complement potential imaging markers for CSVD with cognitive impairment. Due to the small sample size, each case could not cover all the imaging markers. The sample size could affect the statistical power. In particular, no significant difference was observed in the educational level between CSVD and HC groups. Indeed, education years are essential for cognitive disorders. In the present study, there may be no difference in education level due to the small sample size.

There are other limitations in the study. The current study could not correlate imaging features with cognitive scores and alterations of sALFF and dALFF. Moreover, we did not analyze changes in white matter hyperintensity in CSVD with MCI. Furthermore, the superiority verification of the SMS sequence required more samples. Simultaneously, we only exploited functional changes in CSVD with MCI and did not explore the structural network connectivity of the brain. Therefore, future studies should enlarge the sample size to verify the validity of our conclusions. Additionally, big data from multimodality should be leveraged to integrate functional and structural brain activities for understanding the pathological mechanism of CSVD with MCI.

In conclusion, our study observed abnormal intrinsic brain activity patterns of dALFF associated with cognitive decline in CSVD patients. In contrast, abnormal sALFF changes were only identified in CSVD patients with cognitive impairment. The study that combined both sALFF and dALFF may help diagnose CSVD with MCI in the early stage and identify potential abnormal sALFF and dALFF, which is vital for possible early treatment and alleviating disease progression.

## AUTHOR CONTRIBUTIONS

XZ, QH, CX, and XC designed the study. XZ, DZhe, ZW, QW, QY, DZha, XL, YR, SZ,and WT collected the data. XZ analyzed the data and prepared the manuscript. All authors contributed to the article and approved the submitted version.

## CONFLICT OF INTEREST STATEMENT

The authors declare no conflicts of interest.

### PEER REVIEW

The peer review history for this article is available at https://publons.com/publon/10.1002/brb3.3279.

## Supporting information


**TABLE S1** Differences in dynamic ALFF variability among the three groups (160TR).
**Figure S1** Brain regions exhibiting significant differences in dynamic amplitude of fluctuation of low frequency (160TR). **(A)** Brain regions of dynamic amplitude of fluctuation of low frequency showed significant differences among CSVD‐M, CSVD‐W, and HC groups. **(B–D)** Results of post hoc analyses were performed using two‐sample *t*‐tests. Gaussian random field (GRF) corrected. The voxel level and the clustering level were set to *p* < .05 and *p* < .05, respectively. CSVD‐M, cerebral small vessel disease with mild cognitive impairment; CSVD‐W, cerebral small vessel disease without mild cognitive impairment; HC, healthy controls. PoCG, postcentral gyrus; CPL, cerebellum posterior lobe; IPL, inferior parietal lobule; PL, paracentral lobule; FG, fusiform gyrus; R, right; L, left.

## Data Availability

The data analyzed in this study is subject to the following licenses/restrictions: The datasets analyzed in this article are not publicly available. Requests to access the datasets should be directed to Qingling Huang, hql_nju@163.com.
