# Supplementary material for: Aberrant spontaneous static and dynamic amplitude of low‐frequency fluctuations in cerebral small vessel disease with or without mild cognitive impairment
Source: Brain Behav. 2023 Oct 10;13(12):e3279. doi: 10.1002/brb3.3279 (PMC10726894; doi:10.1002/brb3.3279)
Supplement: Supplementary file 1 — TABLE S1 Differences in dynamic ALFF variability among the three groups (160TR). Figure S1 Brain regions exhibiting significant differences in dynamic amplitude of fluctuation of low frequency (160TR). (A) Brain regions of dynamic amplitude of fluctuation of low frequency showed significant differences among CSVD‐M, CSVD‐W, and HC groups. (B–D) Results of post hoc analyses were performed using two‐sample t‐tests. Gaussian random field (GRF) corrected. The voxel level and the clustering level were set to p < .05 and p < .05, respectively. CSVD‐M, cerebral small vessel disease with mild cognitive impairment; CSVD‐W, cerebral small vessel disease without mild cognitive impairment; HC, healthy controls. PoCG, postcentral gyrus; CPL, cerebellum posterior lobe; IPL, inferior parietal lobule; PL, paracentral lobule; FG, fusiform gyrus; R, right; L, left. [file BRB3-13-e3279-s001.docx]

**Methods**

**Diagnostic criteria for CSVD**(Wardlaw et al., 2013; Zanon Zotin, Sveikata, Viswanathan, & Yilmaz, 2021):

MRI images showed any of the following signs: white matter hypersignal, new subcortical infarcts and/or lacunae, cerebral microbleeds, enlarged perivascular Spaces, and cerebral atrophy.

The definitions and imaging standards of these signs were according to the Standards for Reporting Vascular Changes on Neuroimaging (STRIVE) criteria proposed by Wardlaw et al. Lacunae were defined as ovoid or round, subcortical, hypointense cavities with a diameter between 3 and 15 mm on T1 and FLAIR images. New subcortical infarcts were identified as a hyperintense signal on diffusion weighted imaging (DWI) and a reduced apparent diffusion coefficient (ADC) in the corresponding brain region. Moreover, it’s were also defined as acute subcortical lesions in the territory of penetrating arteries, located in the deep grey or white matter of the cerebral hemispheres or brainstem and with a maximum diameter of 20mm in the axial plane on DWI. WMH were described as hyperintensities on FLAIR images, without cavitation. WMHs were assessed using the Fazekas scale on the FLAIR sequence. Cerebral microbleeds are defined as small low signals on the DWI sequence (B=0). The enlarged perivascular space (PVS) is defined as the perivascular extracerebral fluid space, similar to the cerebrospinal fluid signal. Cerebral atrophy is defined as loss of cerebral volume in the upper or subcortical T1 sequence not associated with extensive trauma or infarction. In addition, three-dimensional time-of-flight magnetic resonance angiography (MRA) showed no obvious abnormality.

Possible clinical symptoms: ischemic or hemorrhagic stroke, cognitive decline, mental emotional disorders (indifference, mania), gait disorder, difficulty in urination. The diagnosis was evaluated comprehensively by two senior clinical neuropsychologists.

**Objective memory damage evaluation criteria (**meet any of the following requirements**)**:

A. A neuropsychological associated with episodic memory function scale (including the Auditory Verbal Learning Test, the Rey-Osterrich Complex Figure Test-20 min-delayed recall, and the Logical Memory Test) and other cognitive domain characteristic scale score≤1.0SD.

B. Episodic memory function of two or more related neuropsychological scale score≤1.0 standard deviation (SD).

| **TABLE S1** Differences in dynamic ALFF variability among the three groups(160TR). | | | | | |
| --- | --- | --- | --- | --- | --- |
| **Region(aal)** | Peak MNI coordinate | | | F/T-values | Cluster number |
|  | X | Y | Z |  |  |
| **ANCOVA** |  |  |  |  |  |
| R Cerebellum Posterior Lobe | 24 | -72 | -48 | 7.1617 | 124 |
| L Cerebellum Posterior Lobe | -30 | -60 | -30 | 14.9702 | 301 |
| R Inferior Parietal Lobule | 42 | -42 | 33 | 10.5178 | 229 |
| B Postcentral Gyrus/ Paracentral Lobule | 6 | -42 | 69 | 9.8084 | 387 |
| CSVD-W vs. HC |  |  |  |  |  |
| R Postcentral Gyrus | 39 | -30 | 54 | 4.7330 | 131 |
| CSVD-M vs. HC |  |  |  |  |  |
| R Cerebellum Posterior Lobe | 36 | -63 | -39 | -4.0529 | 117 |
| L Cerebellum Posterior Lobe | -30 | -60 | -30 | -5.2313 | 144 |
| R Inferior Parietal Lobule | 42 | -42 | 33 | -4.8774 | 173 |
| B Paracentral Lobule | 6 | -42 | 69 | 4.2093 | 195 |
| CSVD-M vs. CSVD-W |  |  |  |  |  |
| L Cerebellum Posterior Lobe | -24 | -66 | -33 | -3.9235 | 84 |
| R Inferior Parietal Lobule | 63 | -36 | 24 | -3.6360 | 141 |
| R Postcentral Gyrus | 36 | -33 | 63 | -3.5449 | 96 |
| B Paracentral Lobule | -6 | -36 | 72 | 4.0854 | 89 |
| The x, y, z coordinates are the primary peak locations in the MNI space. ALFF, amplitude of low-frequency fluctuations; HC, healthy controls; CSVD, cerebral small vessel disease; CSVD-W, CVSD without mild cognitive impairment; CSVD-M, CVSD with mild cognitive impairment; L, left; R, right; B, bilateral; MNI, Montreal neurological institute. All results are displayed after adjusting for age, sex and education at a threshold of *p* < 0.05, GRF corrected. | | | | | |

**Figure1S**

Brain regions exhibiting significant differences in dynamic amplitude of fluctuation of low frequency(160TR). **(A)** Brain regions of dynamic amplitude of fluctuation of low frequency showed significant differences among CSVD-M, CSVD-W, and HC groups. **(B,C,D)** Results of post hoc analyses were performed using two-sample t-tests. Gaussian random field (GRF) corrected, The voxel level and the clustering level were set to p＜0.05 and p＜0.05, respectively. CSVD-M, cerebral small vessel disease with mild cognitive impairment; CSVD-W, cerebral small vessel disease without mild cognitive impairment; HC, healthy controls. PoCG, postcentral gyrus; CPL, cerebellum posterior lobe; IPL, inferior parietal lobule; PL, paracentral lobule; FG, fusiform gyrus; R, right; L, left.


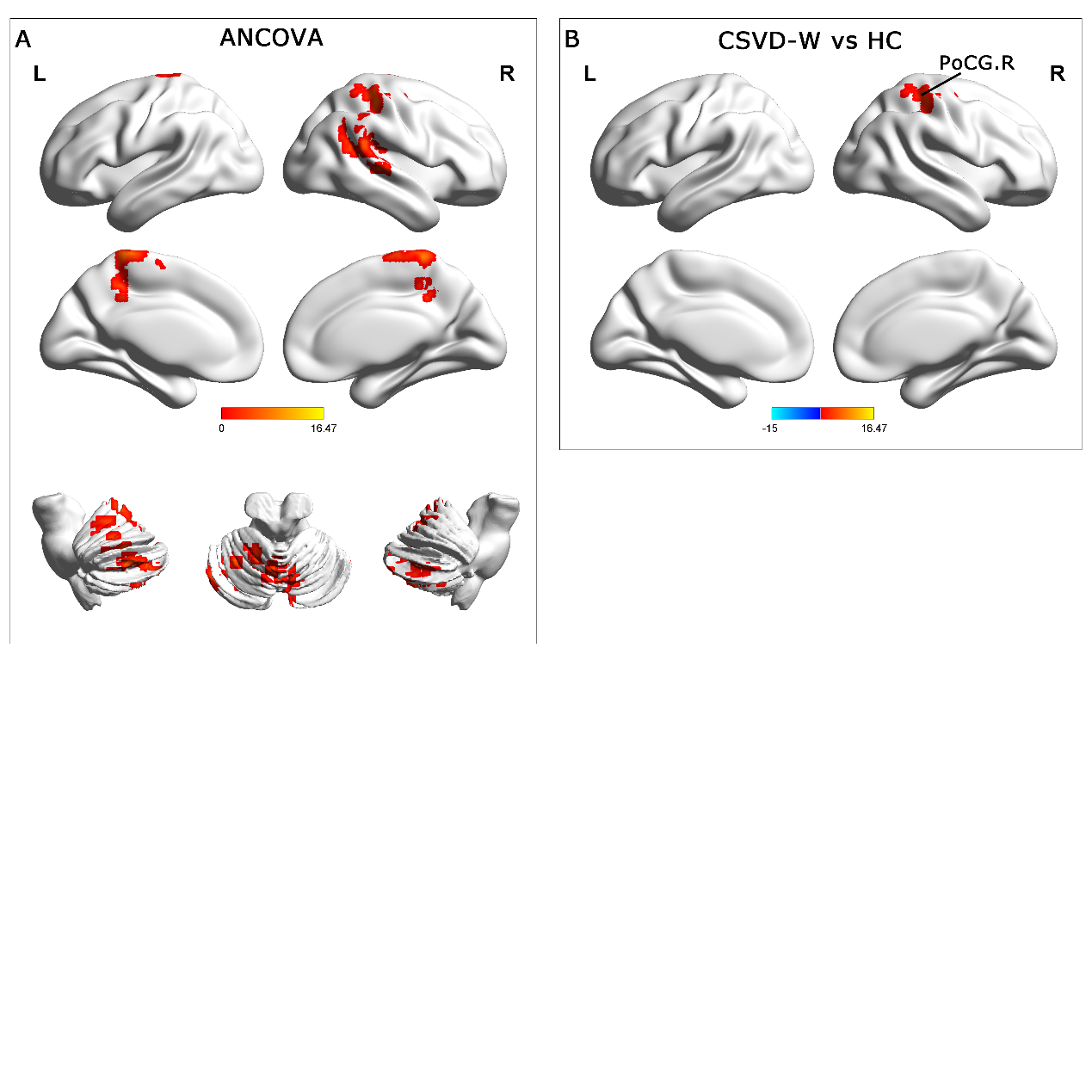


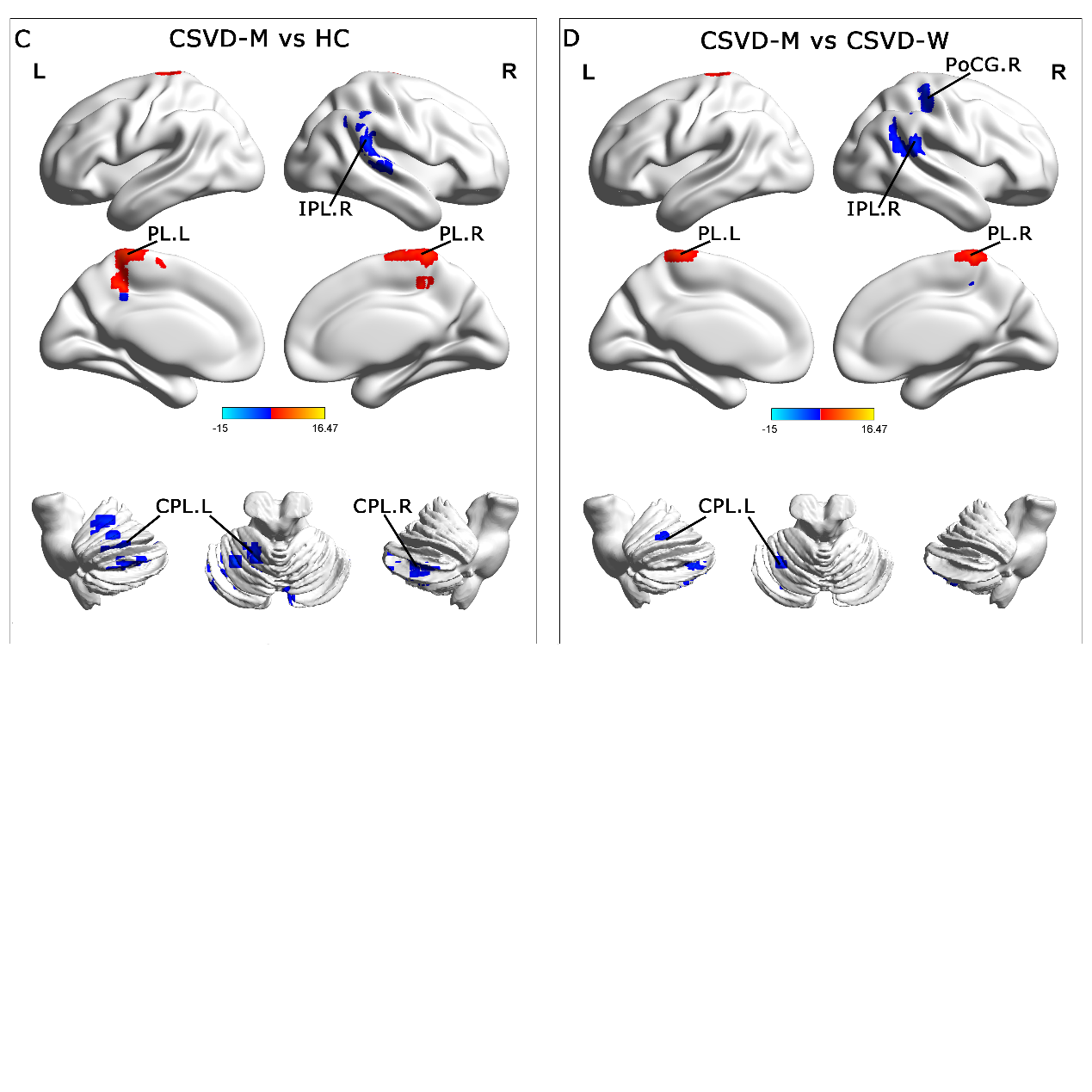


Wardlaw, J. M., Smith, E. E., Biessels, G. J., Cordonnier, C., Fazekas, F., Frayne, R., . . . Dichgans, M. (2013). Neuroimaging standards for research into small vessel disease and its contribution to ageing and neurodegeneration. *Lancet Neurol, 12*(8), 822-838. doi:10.1016/s1474-4422(13)70124-8

Zanon Zotin, M. C., Sveikata, L., Viswanathan, A., & Yilmaz, P. (2021). Cerebral small vessel disease and vascular cognitive impairment: from diagnosis to management. *Curr Opin Neurol, 34*(2), 246-257. doi:10.1097/wco.0000000000000913
